# Bipedal Robot Gait Generation Using Bessel Interpolation

**DOI:** 10.3390/biomimetics9040201

**Published:** 2024-03-28

**Authors:** Zhen Wang, Qingfeng Li, Lei Kou, Danni Zheng, Wende Ke, Dongxin Lu

**Affiliations:** 1 Department of Mechanical and Energy Engineering, Southern University of Science and Technology, Shenzhen 518055, China; z.wang@ubtrobot.com; 2 Health Management System Engineering Center, School of Public Health, Hangzhou Normal University, Hangzhou 311121, China; l20197092@hznu.edu.cn (Q.L.); 2022112011034@stu.hznu.edu.cn (D.Z.); 3 Institute of Oceanographic Instrumentation, Qilu University of Technology (Shandong Academy of Sciences), Qingdao 266075, China; koulei1991@qlu.edu.cn

**Keywords:** Bessel interpolation, inverse kinematics, positioning control, bipedal robot, low stiffness

## Abstract

This paper introduces a novel approach to bipedal robot gait generation by proposing a higher-order form through the parameter equation of first-order Bessel interpolation. The trajectory planning for the bipedal robot, specifically for stepping up or down stairs, is established based on a three-dimensional interpolation equation. The experimental prototype, Roban, is utilized for the study, and the structural sketch of a single leg is presented. The inverse kinematics expression for the leg is derived using kinematic methods. Employing a position control method, the angle information is transmitted to the robot’s joints, enabling the completion of both downstairs simulation experiments and physical experiments with the Roban prototype. The analysis of the experimental process reveals a noticeable phenomenon of hip and ankle joint tilting in the robot. This observation suggests that low-cost bipedal robots driven by servo motors exhibit low stiffness characteristics in their joints.

## 1. Introduction

Bipedal robots, designed to emulate human walking, possess distinctive features, such as a high center of gravity and a discrete distribution of landing points. This makes them a focal point of research in the robotics field. In China, the current robot industry is experiencing rapid growth, and biped robots are increasingly integral to the human-machine society, owing to their humanoid motion capabilities. Particularly, at the consumer level, bipedal robots often utilize servo motors due to their cost-effectiveness and ease of integration. However, the prevalent use of servo motors in bipedal robots introduces challenges associated with low joint stiffness. This results in significant joint errors when executing simple task actions through open-loop control. This paper addresses these challenges by employing the Bessel interpolation method to generate gait trajectories for robots stepping up or down stairs. This approach enhances the robot’s motion performance, enabling it to navigate uneven ground and execute tasks with improved precision and stability.

The evolution of bipedal robots has spurred heightened interest in the research of their gait and footprint planning. Currently, two predominant methods are employed for generating gait trajectories in bipedal robots. One approach involves mimicking human neural reflexes to derive the robot’s gait trajectories. The other mainstream method relies on classical dynamics, wherein gait trajectories are generated by constructing dynamic models or utilizing mathematical interpolation methods. This section focuses on introducing the research content pertaining to the generation of gait trajectories in bipedal robots using the latter, i.e., the classical dynamics-based method. For example, Kajita proposed a linear inverted pendulum (LIPM) model for complex road surfaces in 1991, and its simulation experiments showed that the walking performance of the control method on specific rough terrain is comparable to that of the robot walking on horizontal ground [1]. Subsequently, Kajita combined the linear inverted pendulum model with the ZMP method to apply to a bipedal robot. This method requires the robot to maintain a constant center of mass (COM), and therefore the ASIMO robot bends its knees to move forward [2]. Subsequently, some scholars have improved the algorithm and proposed algorithms such as the Divergent Component of Motion (DCM) algorithm [3], Spatial Quantized Dynamics (SQD) algorithm [4,5,6], the Virtual Mass Ellipsoid Inverted Pendulum (VIP) algorithm [7], etc. Nishiwaki introduced an online method in 2002 that achieves a smooth connection between the subsequent centroid trajectory and the current trajectory by solving the difference equation [8]. Harada used analytical methods in 2004 to smoothly connect COG trajectories to existing trajectories, allowing the robot’s footsteps to change quickly and smoothly [9]. Morisawa improved this method in 2007, allowing robots to adjust their footsteps at any time [10]. In 2014, Samadi also explored the use of the gravity compensated inverted pendulum mode (GCIPM) pattern generation method to generate walking trajectories while ensuring the robot supported the foot movement’s ZMP during stepping [11]. In 2014, Garton detailed the use of a linear inverted pendulum (LIPM) to plan walking trajectories, enabling robots to steadily move forward, stop and restore their direction of motion. He pointed out that the use of a fixed step cycle duration reduces the complexity of mathematics and the amount of computation, thus completing the simplified process of motion planning for the bipedal robot [12]. Imanishi proposed a new bipedal gait control in 2018, which does not rely on time-defined trajectories. The motion rate of the center of mass relative to ZMP is expressed as the gradient of potential. In each step, the potential monotonically decreases from positive to negative, while the positive potential encourages lifting and the negative potential reminds the robot to touch the ground, making the robot’s feet stable and alternating [13]. There are also some other methods for generating gait models, such as using the Hybrid Zero Dynamics (HZD) model [14,15,16]. In addition to model-based methods, spline interpolation can also be used for gait generation, including methods such as cubic and quintic splines [17,18,19,20,21,22]. After gait generation, the Whole Body Control (WBC) and the Model Predictive Control (MPC) methods can be used for robot control [23,24,25,26,27,28,29,30,31].

This paper utilizes the Bessel interpolation method for planning the Roban robot’s gait trajectory. The joint angles are determined through the inverse solution of robot kinematics, and motion execution employs a position control method. The study encompasses both simulation and prototype experiments, with the Roban robot navigating stairs in the V-REP simulation software and a physical prototype experiment simultaneously. The Roban robot, with 12 degrees of freedom in its legs, was provided by China Leju Company. The second section details the establishment of Bessel interpolation, the third section outlines the inverse kinematics solution for the Roban robot, and the fourth section describes the experimental process of the bipedal robot stepping downstairs using Bessel interpolation. The fifth section concludes, a summary of the findings and suggestions for future research studies.

## 2. Inverse Kinematics of Roban Robots

**Figure 1:**
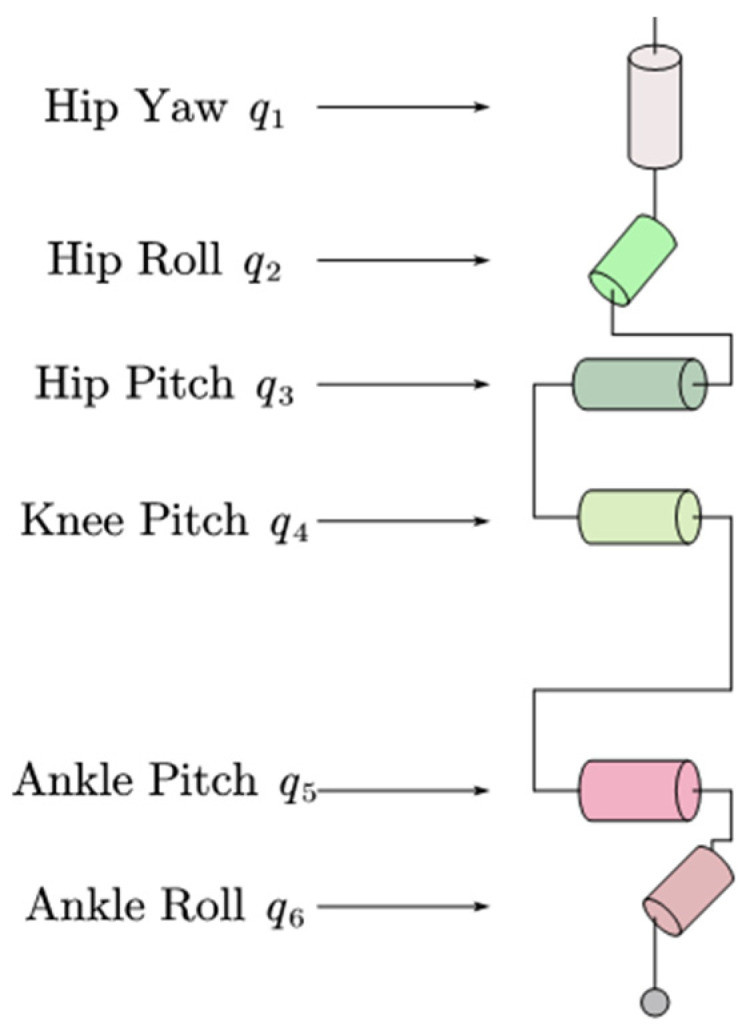
Schematic diagram of the single leg joint of the Roban robot.

Solving the inverse kinematics of a robot involves calculating the angles or movements of each joint based on the robot’s base posture and end configuration. Given that the upper body of the Roban robot functions as a floating base, inverse kinematics calculations are applied to its lower limbs. During low-speed operations, open-loop control is utilized for task execution, and the robot’s joint angle information is derived from its kinematic model. Subsequently, the bipedal robot transmits this angle information to the servo driver through position control, facilitating the execution of the specified task. To obtain the joint angle information for the Roban robot, its legs are modeled using inverse kinematics, as illustrated in Figure 1, depicting the distribution of joint degrees of freedom in a single leg of the Roban robot.

The Roban robot has six degrees of freedom in a single leg, and each joint is equipped with a steering gear. The hip joint is composed of hip yaw, hip roll and hip pitch. The knee joint consists of knee pitch, and the ankle joint consists of an ankle roll and ankle pitch. From top to bottom, the label and angle information of the joint servo are $q_{1}$ to $q_{6}$. The $q_{1}$ joint servo rotates with the vertical line of the ground as its axis; $q_{2}$ and $q_{3}$ are rotated in the forward direction of the robot; $q_{4}$, $q_{5}$, $q_{6}$ are rotated with the lateral axis of the robot. The base coordinate system of the robot is placed on the hip joint. From the perspective of the robot in the forward direction, the forward direction is the *X*-axis, leftward is the *Y*-axis, and vertical upward is the *z*-axis. Therefore, the roll angle is rotated around the *X*-axis, the pitch angle rotates around the *Y*-axis, and the yaw angle is rotated around the *z*-axis. This roll-pitch-yaw way of describing the robot pose is known as the Euler angle method.

**Figure 2:**
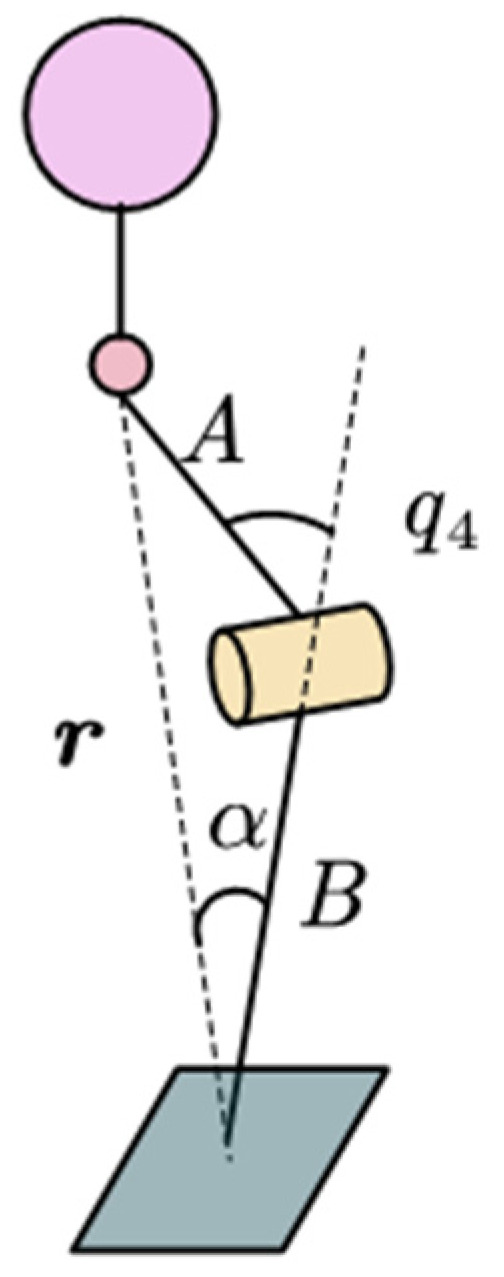
Inverse kinematics of Roban robot knee joint.

When solving the inverse kinematics of the Roban robot, it is first simplified to the triangle shown in Figure 2. A is the thigh length of the Roban robot; B is the leg length of the robot; r is the vector from hip to ankle, and its length is represented by the modulo length C.

The poses of the robot’s torso and feet are ($p_{0}$, $R_{0}$) and ($p_{6}$, $R_{6}$), with the distance from the torso to the hip joint denoted by D, where $p_{i}$ is the position vector and $R_{i}$ is the rotation matrix of each joint’s coordinate system, set to ***E*** and parallel to the world coordinate system, which is defined on the joint cross axis. According to the forward kinematics of the robot, the hip joint position is calculated as follows:

(1) $$p_{1}=p_{0}+R_{0}\left[\begin{array}{l} 0\\ D\\ 0 \end{array}\right]$$

The ***r*** is defined as follows:

(2) $$r=R_{6}^{T}\left(p_{1}-p_{6}\right)\equiv {\left[\begin{array}{ccc} r_{x} & r_{y} & r_{z} \end{array}\right]}^{T}$$

Then the length of *C* is as follows:

(3) $$C=\sqrt{r_{x}^{2}+r_{y}^{2}+r_{z}^{2}}$$

Given the length of *A*, *B* and *C*, *q*_4_ can be written as

(4) $$q_{4}=-\arccos\left(\frac{A^{2}+B^{2}-C^{2}}{2AB}\right)+\pi$$

From the law of cosine, the angle *α* is

(5) $$\alpha =\arccos\left(\frac{B^{2}+C^{2}-A^{2}}{2BC}\right)$$

**Figure 3:**
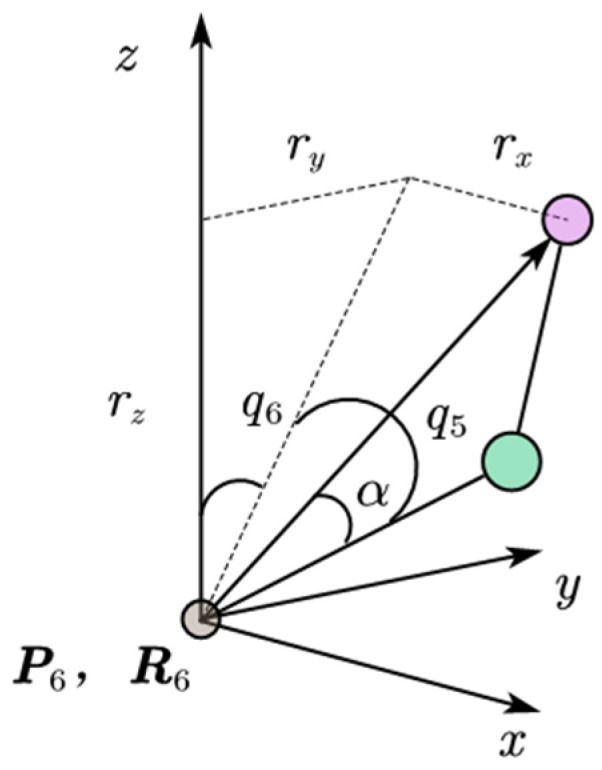
Inverse kinematics of Roban robot ankle joint.

Figure 3 shows the coordinate system of the Roban robot’s single leg ankle joint. The angles of the ankle joint, denoted by $q_{5}$ and  $q_{6}$, can be represented as follows:

(6) $$q_{5}=-\arctan2\left(r_{x},\sqrt{r_{y}^{2}+r_{z}^{2}}\right)-\alpha$$

(7) $$q_{6}=-\arctan2\left(r_{y},r_{z}\right)$$

where *atan*2 is the inverse tangent function.

According to $q_{4}$, $q_{5}$, $q_{6}$ of the robot, $q_{1}$, $q_{2}$, $q_{3}$ are solved. According to the forward kinematics of the robot, the posture of the foot sole can be obtained as follows:

(8) $$R_{f}=R_{1}\left(q_{1}\right)R_{2}\left(q_{2}\right)R_{3}\left(q_{3}\right)R_{4}\left(q_{4}\right)R_{5}\left(q_{5}\right)R_{6}\left(q_{6}\right)$$

The rotation matrix R of the robot is an orthogonal matrix, and its property is

(9) $$RR^{T}=E$$

From Equation (9), Equation (8) can be rewritten as

(10) $$R_{1}\left(q_{1}\right)R_{2}\left(q_{2}\right)R_{3}\left(q_{3}\right)=R_{f}R_{6}^{T}\left(q_{6}\right)R_{5}^{T}\left(q_{5}\right)R_{4}^{T}\left(q_{4}\right)$$

The joint of the Roban robot rotates around roll, pitch and yaw, and the corresponding rotation matrix is the following:

(11) $$R_{x}\left(\phi \right)=\left[\begin{array}{ccc} 1 & 0 & 0\\ 0 & \cos\phi & -\sin\phi \\ 0 & \sin\phi & \cos\phi \end{array}\right]$$

(12) $$R_{y}\left(\theta \right)=\left[\begin{array}{ccc} \cos\theta & 0 & \sin\theta \\ 0 & 1 & 0\\ -\sin\theta & 0 & \cos\theta \end{array}\right]$$

(13) $$R_{z}\left(\psi \right)=\left[\begin{array}{ccc} \cos\psi & -\sin\psi & 0\\ \sin\psi & \cos\psi & 0\\ 0 & 0 & 1 \end{array}\right]$$

Given the angle and rotation axis of the joint steering gear of the Roban robot, denoted by $q_{4}$, $q_{5}$ and $q_{6}$, the right side of Equation (10) is a definite matrix, expressed as

(14) $$R=\left[\begin{array}{ccc} R_{11} & R_{12} & R_{13}\\ R_{21} & R_{22} & R_{23}\\ R_{31} & R_{32} & R_{33} \end{array}\right]$$

From Equation (11) to Equation (13), the expression of $q_{1}$, $q_{2}$ and $q_{3}$ can be obtained as follows:

(15) $$\left[\begin{array}{ccc} c_{1}c_{3}-s_{1}s_{2}s_{3} & -s_{1}c_{2} & c_{1}s_{3}+s_{1}s_{2}c_{3}\\ s_{1}c_{3}+c_{1}s_{2}s_{3} & c_{1}c_{2} & s_{1}s_{3}-c_{1}s_{2}c_{3}\\ -c_{2}s_{3} & s_{2} & c_{2}c_{3} \end{array}\right]$$

Thus,

(16) $$\left[\begin{array}{ccc} c_{1}c_{3}-s_{1}s_{2}s_{3} & -s_{1}c_{2} & c_{1}s_{3}+s_{1}s_{2}c_{3}\\ s_{1}c_{3}+c_{1}s_{2}s_{3} & c_{1}c_{2} & s_{1}s_{3}-c_{1}s_{2}c_{3}\\ -c_{2}s_{3} & s_{2} & c_{2}c_{3} \end{array}\right]=\left[\begin{array}{ccc} R_{11} & R_{12} & R_{13}\\ R_{21} & R_{22} & R_{23}\\ R_{31} & R_{32} & R_{33} \end{array}\right]$$

Among them, by analogy with $c_{2}\equiv \cos q_{2}$ and  $s_{2}\equiv \sin q_{2}$, the remaining identity meanings can be obtained. Finally, the expressions of $q_{1}$, $q_{2}$ and $q_{3}$ can be obtained as follows:

(17) $$q_{1}=\arctan2\left({-R}_{12},R_{22}\right)$$

(18) $$q_{2}=\arctan2\left(R_{32},{-R}_{12}s_{1}+R_{22}c_{1}\right)$$

(19) $$q_{3}=\arctan2\left({-R}_{31},R_{33}\right)$$

At this point, the inverse kinematics expression of the Roban robot is solved.

## 3. Bessel Interpolation Method

Discontinuous gait trajectories in robots can lead to infinite acceleration and rigid impacts, posing a risk of damage due to the resulting torque on joint actuators. To avoid such issues, bipedal robots must adhere to smooth trajectories during walking tasks. Currently, gait modeling based on 3D linear inverted pendulum or Zero Moment Point (ZMP) methods is prevalent. These methods are mutually inverse in terms of input and output. When navigating designated slope step ground, Z-direction trajectory interpolation is necessary for bipedal robots using these approaches. Alternatively, bipedal robots can employ the Virtual Mass Ellipsoid Inverted Pendulum (VIP) method to handle non-planar problems, typically requiring computer-based solutions. However, for low-cost robot prototypes engaged in low-speed motion, the reliance on computer performance is minimized. Thus, this section adopts the Bessel interpolation method for generating the gait trajectory of the bipedal robot, focusing on the generation process in detail.

### 3.1. Use and Characteristics of Bezier Curve

Bezier curve was originally an application curve of two-dimensional figure. Its mathematical expression was originally derived from Bernstein polynomials, which proved that continuous function within the [a, b] interval can be approximated by polynomials, and the approximated functions have strong convergence. For a continuous function, multiple Bernstein polynomials can be selected. When the order of Bernstein polynomials approaches infinity, the polynomial will converge to the original function. Later, French mathematician Casteljau graphically processed the polynomial and proposed a corresponding numerical solution algorithm to generate a fitted two-dimensional graphical curve. Pierre Bezier, a French engineer, used this method to complete the industrial design of the auxiliary car body and spread it widely. Therefore, this method is called the Bessel interpolation method. At present, this method can not only fit two-dimensional curves but also complete curve fitting in space. It includes three types of sampling points, namely: the starting point, the control point and the ending point. The Bezier curve can be changed by adjusting the control points, and the curvature of the Bezier curve can be adjusted. The method of changing the curvature of the Bezier curve by adjusting the control points is very important for the gait adjustment of subsequent robots. The Bessel interpolation method is now used to complete the design of robots’ downstairs gait trajectory.

**Figure 4:**
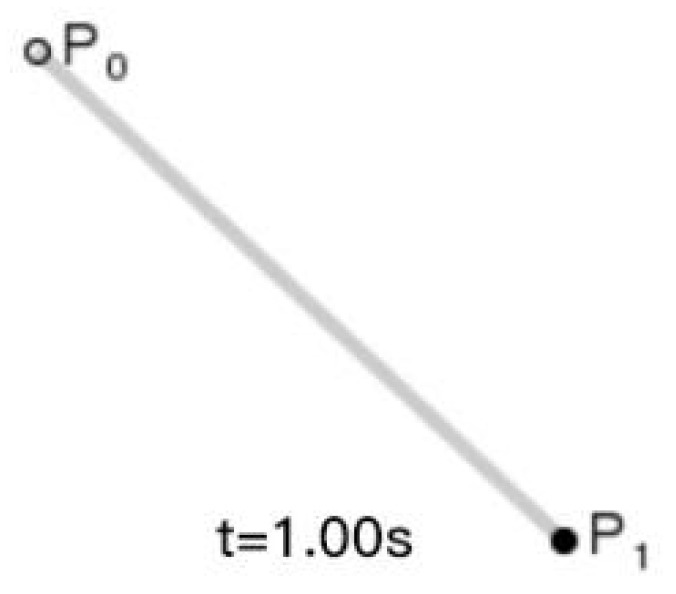
First order Bessel line segment.

The Bezier curve can be constructed by setting sampling points, which include the starting point, the ending point and the control point. By adjusting the number of control points, the degree of the Bezier curve can be adjusted. For example, a first-order Bezier curve is composed of two points, 0 control points, and the fitted Bezier curve is a first-order straight line. Its expression is as follows:

(20) $$B\left(t\right)=\left(1-t\right)P_{0}+tP_{1},\;\;\;t\in \left[0,1\right]$$

where *P*_0_ and *P*_1_ are the start and end points of the Bezier curve, and *t* is the parameter, whose curves are shown in Figure 4.

When the control point is 1, the fitted Bezier curve is a second-order curve, and its expression is as follows:

(21) $$B\left(t\right)={\left(1-t\right)}^{2}P_{0}+2t\left(1-t\right)P_{1}+t^{2}P_{2},t\in \left[0,1\right]$$

**Figure 5:**
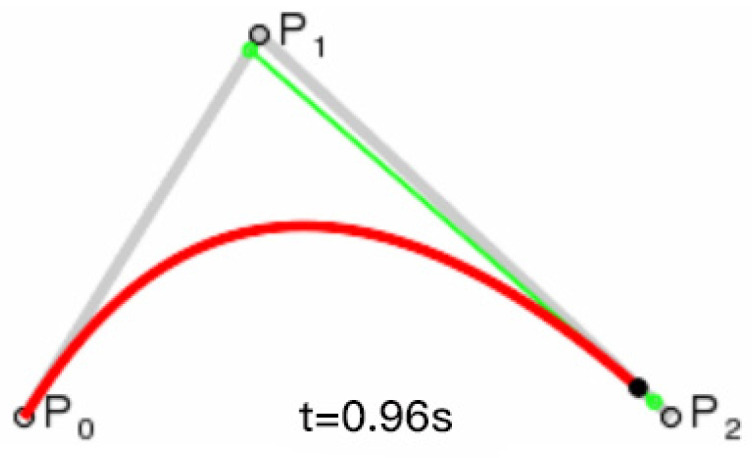
Second order Bezier curve.

The $P_{0}$ and $P_{2}$ are the starting and ending points of the Bezier curve, and $P_{1}$ is the control point. The final expression is a conic, as shown in Figure 5. The Bezier curves for the remaining orders are followed in the same way.

The Bezier curve exhibits three key characteristics: it passes through both the starting and ending points and is tangent to the feature line at both ends. The control point influences the curvature of the Bezier curve by pulling the fitted curve toward the current control point, providing convenient adjustment of the curve’s radian. Planar control points play a crucial role in shaping the curve, as altering their coordinates directly impacts the fitted Bezier curve’s form. The Bezier curve lies within the convex hull of the feature polygon, offering a smoother representation compared to the polygon.

Widely employed in computer graphics, the Bezier curve is created and edited with simple starting points, ending points and control points. Adjusting the starting and ending points alters the position of the Bezier curve in the world coordinate system, while changing the control points influences the curvature to meet the requirements of bipedal robots. For instance, when modifying the Bezier curve trajectory of a bipedal robot’s swing leg, it allows for the adjustment of the swing leg’s stability during the swinging process. Due to their capacity for easy adjustment of sample points, Bezier curves serve as an ideal tool for graphic editing and creation, being utilized by engineers, academics and other professionals.

### 3.2. Higher Order Bezier Curve Fitting Method

The order of the Bezier curve varies with the number of control points. As mentioned in the previous section, when the Bezier curve has zero control points, the Bezier curve is a first-order line segment. When the Bezier curve has one control point, the Bezier curve is a second-order quadratic curve and so on. The mathematical formulas of the first and second order curves are given in the above subsections. In the following, the expression of the second order and the formula of the higher order Bezier curve are derived from the first order formula.

The first-order Bezier curve has two points, namely the points $P_{0}$ and $P_{1}$, and its parametric equation can be expressed as Equation (20).

The quadratic Bezier curve has three points, respectively, $P_{0}$, $P_{1}$ and $P_{2}$, where $P_{0}^{\prime }$ and $P_{1}^{\prime }$, respectively, are fixed points on a line *P*_0_*P*_1_ and *P*_1_*P*_2_. According to the first-order Bezier curve, the parametric equations of $P_{0}^{\prime }$ and $P_{1}^{\prime }$ are as follows:

(22) $$P_{0}^{\prime }=\left(1-t\right)P_{0}+tP_{1}$$

(23) $$P_{1}^{\prime }=\left(1-t\right)P_{1}+tP_{2}$$

By fitting the parametric equations of $P_{0}^{\prime }$ and $P_{1}^{\prime }$ according to the first-order equation, we can get the following:

(24) $$\begin{array}{ll} B_{2}\left(t\right) & =\left(1-t\right)P_{0}^{\prime }+tP_{1}^{\prime }\\ & =\left(1-t\right)\left(\left(1-t\right)P_{0}+tP_{1}\right)+t\left(\left(1-t\right)P_{1}+tP_{2}\right)\\ & ={\left(1-t\right)}^{2}P_{0}+2t\left(1-t\right)P_{1}+t^{2}P_{2} \end{array}$$

For the expression of the Bezier curve of higher order, suppose that $p_{k}^{n}$ is the coefficient of the *k*th point of the Bezier curve of order. In the $B_{n-1}\left(t\right)$, by utilizing the second order Bessel method based on *P_k_* in $\left(1-t\right)P_{k}^{\prime }+tP_{k+1}^{\prime }$, will $P_{k-1}$ transition to $\left(1-t\right)P_{k-1}^{\prime }+tP_{k}^{\prime }$. In the function *B_n_*(*t*), in the presence of a new parameter, $P_{k}^{\prime }$, it can be expressed as $P_{k}^{\mathrm{prime}}=\left(1-t\right)P_{k}+tP_{k-1}$.

The following condition needs to exist:

(25) $$\left\{\begin{array}{c} p_{i}\;\;\;\;n=1\\ p_{k}^{n}=\left(1-t\right)p_{k}^{n-1}+tp_{k-1}^{n-1},\;\;\;\;n=1,2,\ldots ,m,\;\;\;\;k=0,1\ldots ,n-1 \end{array}\right.$$

to prove the following equation:

(26) $$p_{k}^{n}=\left(\begin{array}{c} n\\ k \end{array}\right)t^{k}{\left(1-t\right)}^{n-k}$$

(27) $$\begin{array}{ll} p_{k}^{m+1} & =\left(1-t\right)p_{k}^{m}+tp_{k-1}^{m}\\ & =\left(\begin{array}{l} m\\ k \end{array}\right)t^{k}{\left(1-t\right)}^{m+1-k}+\left(\begin{array}{l} m\\ k-1 \end{array}\right)t^{k}{\left(1-t\right)}^{m+1-k}\\ & =\left(\begin{array}{l} m+1\\ k \end{array}\right)t^{k}{\left(1-t\right)}^{m+1-k} \end{array}$$

When n=m+1, Equation (27) conforms to the law of Equation (26), and the result still holds. Thus, the induction hypothesis holds and the Equation (28) for Bezier curves of any order *n* is obtained. Using this formula, the gait trajectory of a robot based on the Bezier method can be generated.

(28) $$B_{n}\left(t\right)=\sum_{k=0}^{n-1}p\begin{array}{c} n\\ k \end{array}P_{k}=\sum_{k=0}^{n-1}\left(\begin{array}{c} n\\ k \end{array}\right)t^{k}{\left(1-t\right)}^{n-k}P_{k}$$

**Figure 6:**
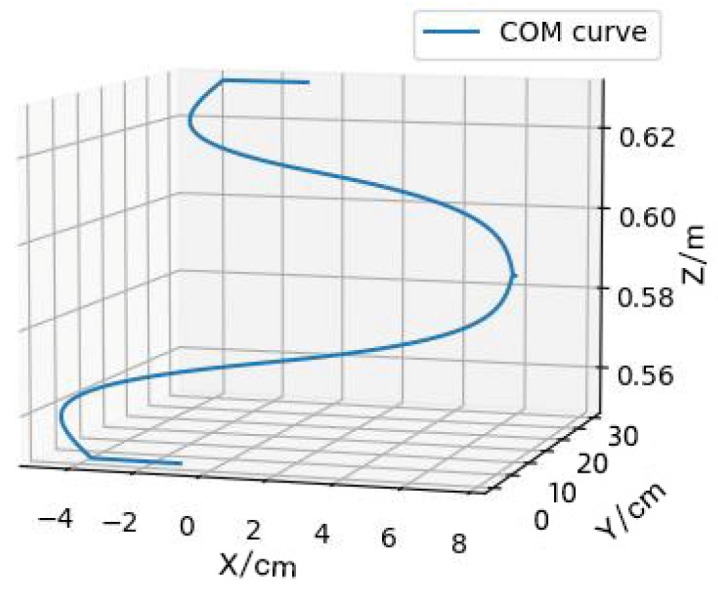
The Bezier curve for the center of mass.

According to this method and based on specific parameters, such as the robot’s centroid position, step height, step length, foot length and so on, the Bezier curve of the robot centroid is planned and generated, as shown in Figure 6.

## 4. Experiments

The Roban robot underwent stair simulation experiments using Virtual Robot Experimentation Platform (V-REP) software, alongside completing a physical prototype experiment of stepping downstairs. The experimental results validate the efficacy of the Bessel interpolation-based gait generation method and shed light on the specific motion characteristics of low-cost bipedal robots under open-loop control. These findings enhance our understanding of the practicality of the proposed approach in real-world scenarios.

### 4.1. The Bipedal Roban Robot

**Figure 7:**
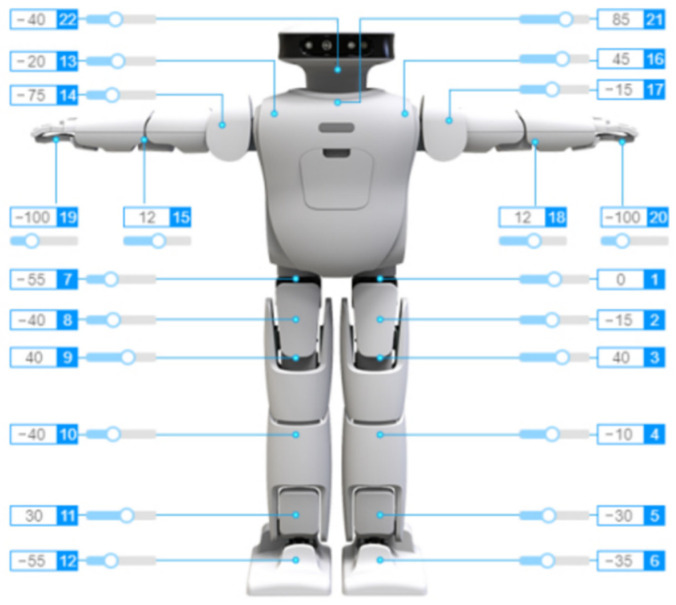
Roban robot and its degree of freedom allocation.

This experimental platform utilizes the bipedal robot prototype, Roban, provided by Leju (Shenzhen) Robot Technology Co., Ltd., Shenzhen, China. The prototype serves as an AI display and application platform, based on ROS, for higher education institutions, research institutes and other scientific research organizations. It boasts characteristics such as open-source architecture and scalability. Equipped with a depth camera, a built-in V-SLAM vision algorithm and compatibility with platforms such as UCF and Microsoft, it excels in intelligent operations such as speech recognition, SLAM-based indoor navigation mapping, walking along curves and executing movements on slopes and stairs. Leju offers a comprehensive set of educational programs for artificial intelligence talents, covering topics ranging from programming and robot motion control to computer vision. These programs are suitable for independent learning by students majoring in mechanical, computer, automation and related disciplines. Figure 7 illustrates the Roban robot, providing details on the distribution of its degrees of freedom and the zero offset for each joint.

The remaining content is shown in Table 1.

### 4.2. Simulation Experiment

The Bessel interpolation method falls under the category of static gait trajectory generation. When the robot employs position control, the Bessel interpolation method is effective in generating the low-speed gait required for descending stairs. Considering the hardware parameters of the Roban robot, the chosen steps for the experiment are 50 cm in length, 15 cm in width and 4 cm in height.

**Figure 8:**
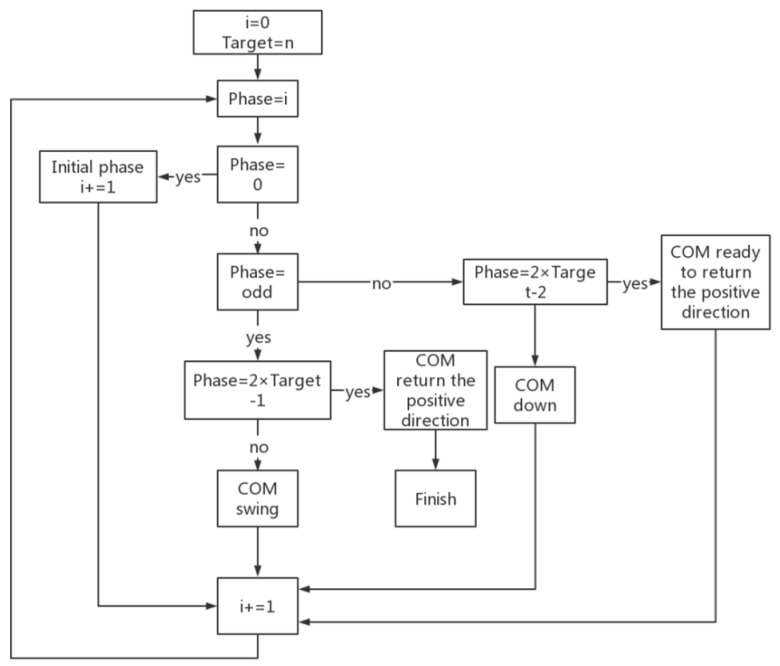
Process framework of Roban robot stepping downstairs based on Bezier method.

The stair-stepping simulation experiment is conducted in V-REP after establishing the step simulation environment. Initialization tasks, including ROS and joint variable initialization, are undertaken. ROS-based publisher and subscriber are defined to facilitate information exchange. The robot executes motion actions based on Bezier curves using position control. Starting from the initial standing position, the robot smoothly navigates the stair descent process, employing Bezier interpolation to generate its trajectory in each phase of the experiment. The phases include the following: phase = 0, phase = odd, phase = even, phase = 2 × Target (the number of Target stairs)-2, phase = 2 × Target-1. The complete process of going down the stairs can be summarized by the flow chart shown in Figure 8.

Phase = 0 is the initial descent phase of the robot on the stairs. During this phase, the robot completes the left swing of the hip joint, so that its center of mass is closer to the left, and initiates the swing of its right leg.

Phase = odd is the swing phase of the robot’s center of mass during the process of stepping downstairs; for example, when phase = 1, the robot has just completed the descent of one step, and its center of mass is on the left. If the robot lifts its left foot at this time, the robot will fall down. Therefore, the center of mass of the robot is set to move to the right, and this stage is the center of mass translation stage.

In even phases (phase = even), the center of mass of the robot descends the stairs during the process of going down the stairs. For example, when phase = 2, the robot completes the change of the center of mass. The robot lifts its left leg and completes the descent process of two steps; this phase is the centroid dropping phase of the robot.

Phase = 2 × Target-2 is the phase when the robot is ready to return to the positive direction; for example, the robot’s target falls to the third step, that is, Target = 3. When the phase = 4, the swing leg of the robot completes the descent of one step. Both of its feet should finally land on the same step, marking this phase as the foot recovery phase of the robot.

Phase = 2 × Target-1 is the phase when the robot returns to the upright position; for example, at phase = 5, the robot’s feet return to the upright position because the mass is centered on the left. To make the robot stand completely straight, the center of mass of the robot is translated to the right. This stage is the center of mass of the robot back to the positive stage.

**Figure 9:**
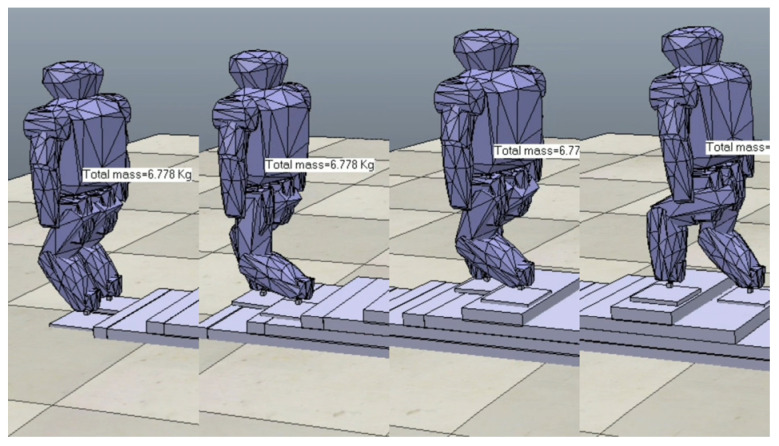
Simulation of Roban stepping downstairs (from right to left).

According to the above design process and step parameters, the descent trajectory of a bipedal robot based on the Bezier curve is designed. The Roban robot generates the position information transmitted to the joint servo through its inverse kinematics expression, and it can successfully perform the downstairs task on the V-REP simulation platform, based on ROS, as shown in Figure 9.

### 4.3. Prototype Experiment

When the simulation code is fully utilized in the prototype’s downstairs experiment, the results prove less than ideal. Through adjustments to the Bezier curve’s curvature and the time period for the servo to accept position commands, the Roban prototype achieves successful stair-stepping. Influential input parameters for Bezier curvature include the robot’s centroid position, step height, step length and foot length. Despite these parameters being determined in the physical world, the robot’s inherent errors necessitate further adjustments to execute the stair-stepping task based on Bezier curves. Several considerations are essential during this adjustment phase:

(1) Parameters for adjusting the robot’s position:

Adapting the robot’s center of mass prevents forward or backward falls during leg swings.

Altering the trajectory of the robot’s legs addresses issues with the foot falling prematurely before lifting off the steps.

Adjusting the control point of the trajectory affects the position and swing inertia of the robot’s swing foot.

Continuous trajectory adjustments are crucial to prevent robot shaking or instability during stair descent.

(2) Adjustment of the robot’s time cycle parameters:

Given that this static gait method suits slow descents, an overly fast overall speed introduces significant inertia during leg swings and center of mass translation.

Increasing the running time of the overall robot reduces inertia, preventing forward or backward falls.

(3) Parameters for adjusting the robot’s step height:

Similar to adapting the trajectory of the robot’s legs, this parameter prevents the robot’s feet from falling prematurely before lifting off the step plane.

(4) Parameters for adjusting the robot’s step length and foot length:

To address the accumulated errors observed during the stair descent process, adjusting these parameters helps maintain the intended distance for each step.

**Figure 10:**
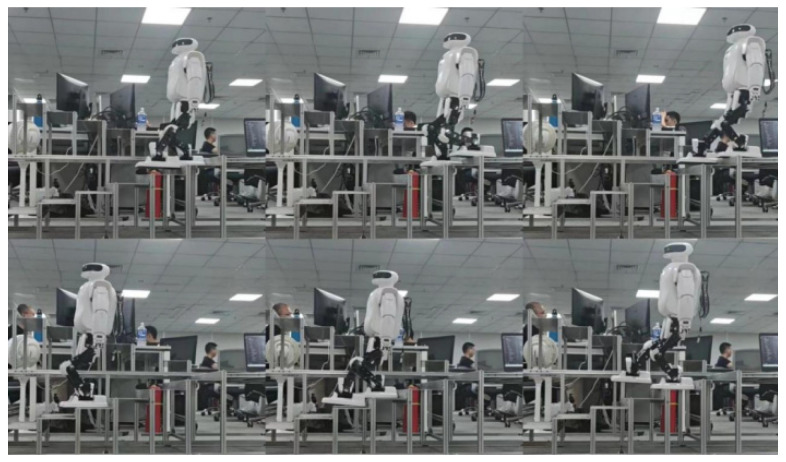
Roban stepping downstairs (from right to left).

Following the meticulous adjustment of these parameters, the Roban robot successfully executes the stair-stepping process in the real world, as illustrated in Figure 10.

Examining the Roban robot’s stair descent process in the figure above reveals significant errors, including jitter, hip joint tilt, backward tilt and misalignment of the ankle joint with the step surface. These issues stem not only from limitations in the static gait method but also from various errors within the Roban prototype itself, such as servo installation discrepancies, manufacturing inaccuracies, and the inherent low-bending stiffness of gears. These factors collectively contribute to insufficient joint stiffness in both the hip and ankle joints of the bipedal robot. Addressing these challenges requires ongoing refinement in both gait generation methods and mechanical components to enhance overall performance and stability during complex tasks like stair descent.

## 5. Conclusions

This paper introduces the Bezier interpolation method for generating the gait trajectory of the Roban robot during stair descent. The inverse kinematics expression of the robot is solved, and the Bezier gait curve is successfully applied to both the staircase simulation and prototype experiments using position control. The analysis of the experiment video reveals that the Roban robot exhibits jitter, hip joint tilt, backward tilt and an ankle joint misalignment with the step surface. These observations are attributed to the low joint stiffness of the Roban robot. Despite these challenges, this research lays the groundwork for improving the motion performance of low-cost bipedal robots, offering valuable insights for future development.

## Figures and Tables

**Table 1:** Specific parameters of Roban robot.

DOF	22
Single leg DOF	6
Mass/kg	6.6
Height/cm	70

## Data Availability

The data presented in this study are available on request from the corresponding author. The data are not publicly available, as they are only accessible to teams interested in collaboration.
